# FEELING AND LIVING WELL: NETWORK COMPOSITION AND PREDICTING SELF-RATED HEALTH FOR RACIAL AND ETHNIC MINORITIES

**DOI:** 10.1093/geroni/igz038.633

**Published:** 2019-11-08

**Authors:** Eli X Ornelas

**Affiliations:** University of Nebraska-Lincoln, Lincoln, Nebraska, United States

## Abstract

Racial and ethnic inequities in health among older adults in the U.S. are well documented. A substantial amount of the health literature focuses on physical and mental disparities; however, less research has examined racial and ethnic differentials in subjective, self-rated health (SRH). Prior research has documented racial and ethnic inequities in SRH, though mechanisms by which these disparities occur are still largely unknown. One potential mechanism by which these disparities may arise is through unequal access to psychosocial resources through variability in social networks. Utilizing data from the 2006 Health and Retirement Study (HRS) along with the 2006 HRS Psychosocial and Lifestyle Questionnaire, the current study seeks to explore racial and ethnic differentials in SRH from a social network perspective. Ordinal logistic regression is used to predict SRH by race and Hispanic ethnicity potentially mediated by possession and number of friends, frequency of contact with friends, and psychosocial and subjective well-being. Results indicate that older black adults are less likely to rate their health in a higher category of SRH than older white adults, and this relationship is not significant for other racial groups and Hispanics. Additionally, psychosocial and subjective well-being and frequent written-communication with friends are found to significantly predict better SRH. The results suggest that feeling well mentally is crucial for SRH, but equally important is regular contact with friends. Further research should employ more robust measures of social networks to elucidate the role that network composition plays in predicting SRH for racial and ethnic minorities.

